# The Effects of Bougie Diameters on Tissue Oxygen Levels After Sleeve Gastrectomy: A Randomized Experimental Trial

**DOI:** 10.4274/balkanmedj.2017.0484

**Published:** 2018-05-22

**Authors:** Can Konca, Ali Abbas Yılmaz, Süleyman Utku Çelik, Selami Ilgaz Kayılıoğlu, Özge Tuğçe Paşaoğlu, Halil Arda Ceylan, Volkan Genç

**Affiliations:** 1 Department of Surgery, Ankara University School of Medicine, Ankara, Turkey; 2 Department of Anesthesiology and Reanimation, Ankara University School of Medicine, Ankara, Turkey; 3 Department of Surgery, University of Health Sciences, Ankara Numune Training and Research Hospital, Ankara, Turkey; 4 Department of Medical Biochemistry, Gazi University School of Medicine, Ankara, Turkey; 5 Department of Internal Medicine, Ankara University School of Medicine, Ankara, Turkey

**Keywords:** Animal study, bariatric surgeries, bougie diameter, gastric leak, randomization, wound healing

## Abstract

**Background:**

Staple-line leak is the most frightening complication of laparoscopic sleeve gastrectomy and several predisposing factors such as using improper staple sizes regardless of gastric wall thickness, narrower bougie diameter and ischemia of the staple line are asserted.

**Aims:**

To evaluate the effects of different bougie diameters on tissue oxygen partial pressure at the esophagogastric junction after sleeve gastrectomy.

**Study Design:**

A randomized and controlled animal experiment with 1:1:1:1 allocation ratio.

**Methods:**

Thirty-two male Wistar Albino rats were randomly divided into 4 groups of 8 each. While 12-Fr bougies were used in groups 1 and 3, 8-Fr bougies were used in groups 2 and 4. Fibrin sealant application was also carried out around the gastrectomy line after sleeve gastrectomy in groups 3 and 4. Burst pressure of gastrectomy line, tissue oxygen partial pressure and hydroxyproline levels at the esophagogastric junction were measured and compared among groups.

**Results:**

Mortality was detected in 2 out of 32 rats (6.25%) and one of them was in group 2 and the cause of this mortality was gastric leak. Gastric leak was detected in 2 out of 32 rats (6.25%). There was no significant difference in terms of burst pressures, tissue oxygen partial pressure and tissue hydroxyproline levels among the 4 groups.

**Conclusion:**

The use of narrower bougie along with fibrin sealant has not had a negative effect on tissue perfusion and wound healing.

Obesity is a pandemic condition and a major health problem in both developed and developing countries. According to the World Health Organization data, 39% and 13% of adults aged 18 years and over are overweight and obese, respectively (1). Laparoscopic sleeve gastrectomy (LSG) is one of the most popular techniques used for weight loss in several countries, including the USA (2). LSG was initially advocated as the first step of a two-staged procedure for high-risk super-obese patients (3). In recent years, LSG has become a popular procedure, and its resulting weight loss and comorbidity resolution are similar to those of laparoscopic gastric bypass (4,5,6). Staple line leak is one of the most frequent and serious complications of LSG. Gastric leak rates are reported to be around 0%-20% in different studies (7,8), and the location of the leak in the vast majority of cases (92%) is the proximal region of the esophagogastric junction (EGJ) (7). However, the risk of staple line leak is of great concern and needs further investigation. In literature, several predisposing factors of staple line leak include using improper staple sizes regardless of gastric wall thickness, narrow bougie diameter, and ischemia of the staple line (7,9). Thus far, no quantitative study has been conducted on the relationship between staple line leak and tissue ischemia. Hence, this study aims to evaluate the effects of different bougie diameters on tissue oxygen partial pressure (PtO_2_) at the EGJ after sleeve gastrectomy.

## MATERIALS AND METHODS

### Animals, experimental protocol, and study design

Thirty-two male Wistar albino rats, with an average weight of 317±9.4 g and aged 8 months, were used in this study. The animals were randomly divided into four groups, with eight rats each. Group 1 underwent sleeve gastrectomy via 12-Fr bougie; group 2 underwent sleeve gastrectomy via 8-Fr bougie; group 3 underwent sleeve gastrectomy via 12-Fr bougie followed by fibrin sealant (Tisseel^®^ fibrin sealant; Baxter, Deerfield, Illinois, USA) application around the gastrectomy line; and group 4 underwent sleeve gastrectomy via 8-Fr bougie followed by fibrin sealant application around the gastrectomy line. The study was designed as a randomized and controlled animal trial with 1:1:1:1 allocation ratio and performed in the Animal Laboratory of Ankara University School of Medicine in Ankara, Turkey. The computer-generated random sequences of numbers were used for randomization.

### Ethics statement

The study was approved by the Animal Experiments Ethics Committee in Ankara University (Approval number: 13-11-79) in concordance with the Guide for the Care and Use of Laboratory Animals published by the US National Institutes of Health.

### Surgical technique

The operations were performed under heat lamps to maintain the body temperature within 35 °C-36 °C and by the same surgeon conductive keratoplasty with the same technique. Anesthesia was attained by intramuscular injection of 40 mg/kg body weight ketamine (Ketalar^®^; Parke-Davis, Eczacıbaşı, İstanbul, Turkey) and 5 mg/kg body weight xylazine (Rompun^®^; Bayer Türk, İstanbul, Turkey). The subjects respired spontaneously during the operation, and 12-hour day/12-hour night cycles were provided. Laparotomy was performed with a 3 cm midline incision following cleaning with 10% povidone–iodine. After dissection of the greater curvature by using 4/0 silk sutures (Sterisilk^®^; SSM, İstanbul, Turkey), gastrotomy was performed 5 mm proximal to the pylori, and the gastric contents were aspirated. An aspiration catheter (as a bougie) was placed aside for lesser curvature through the gastrotomy. With the guidance of the bougie, a bulldog clamp was placed on the stomach longitudinally, and sleeve gastrectomy was performed over the clamp. The gastrectomy line was closed with double-layer continuous technique using 6/0 Prolene^®^ sutures (Ethicon US, Cleveland, Ohio, USA). The gastrotomy aperture was sutured similarly after the removal of the bougie, and the abdominal fascia and skin were closed with continuous 3/0 silk sutures (Sterisilk^®^; SSM, İstanbul, Turkey). The aspiration catheters were used as bougies. Catheters with 12-Fr diameter (Bıçakçılar, İstanbul, Turkey) were used in groups 1 and 3, and those with 8-Fr diameter (Kaishou, Changzhou, Jiangsu, China) were used in groups 2 and 4. Fibrin sealant was applied around the gastrectomy line of subjects in groups 3 and 4.

### Postoperative care

Hydration of all subjects was sustained by injecting 3 mL of 0.9% NaCl and 3 mL of 5% dextrose subcutaneously right after the surgery and every 12 hours until postoperative second day. Two milligrams of meloxicam per kilogram was also administered for analgesia. All subjects were monitored under heat lamps until they recovered from anesthesia. All subjects were also observed without oral intake on the day of surgery. On the 1^st^ and 2^nd^ postoperative days, water and Ensure Plus^®^ Vanilla (Abbott, Illınois, USA) were administered ad libitum. Standard rat diet was given to all subjects on the 3^rd^ postoperative day.

### Measurements

The Licox^®^ monitoring system (Integra Life Sciences Corp., San Diego, California, USA) and 0.6 mm Clark type polarographic microprobes (Licox^®^ CMP cc1, Integra Life Sciences Corp., San Diego, California, USA) were used to determine tissue PtO_2_. His angle of the stomach was identified without impairing its vascular supply. The first PtO_2_ measurement (PtO_2_-0) was carried out immediately after laparotomy. PtO_2_ was measured (PtO_2_-1) again in the remnant of the angle of his right after suturing of the gastrectomy line in groups 1 and 2 and right after applying the fibrin sealant in groups 3 and 4. The second laparotomy was performed in all groups on the 30th day. The stomach was dissected without impairing its vascular supply, and PtO_2_ was measured (PtO_2_-30) in the remnant of the angle of his. The stomach was subsequently dissected from adjacencies, and the segment between the distal esophagus and the duodenum was removed totally. The subjects were then sacrificed by administering a high dose of thiopental sodium. The stomach was irrigated, and burst pressure of the gastrectomy line was measured according to the technique implemented by Kuzu et al. (10). A catheter was introduced and fixed with 2/0 silk thread from the duodenum. The distal esophageal end was ligated with 2/0 silk thread to close the lumen. A three-way circuit was established comprising the catheter, the registered gauge, and the arterial blood pressure monitoring system. A flow of 2 mL/min was injected into the circuit until the rupture of the gastrectomy line, and the maximum pressure (mmHg) was recorded at the time of the rupture. Afterwards, a piece of tissue including the EGJ and the proximal gastrectomy line was resected and gently washed. The tissue samples were placed in cryogenic tubes (Cryo.S™, Greiner Bio-One, Frickenhausen, Germany) and kept frozen at -80 °C until the day of hydroxyproline measurement. Hydroxyproline measurement was conducted using a previously described method (11). The principle of the method is the oxidation of hydroxyproline in the samples with chloramine T and the reaction of the oxidized molecule with Erlich reagent to yield a chromophore compound, which can be measured spectrophotometrically. The tissues were weighed and digested with hydrochloric acid in a digestion oven. After the acid digestion, a particular amount of the samples were obtained and placed in a desiccator to allow the liquid to evaporate. The samples were then redissolved in isopropyl alcohol and added with chloramine T and Erlich reagent. After the incubation period, absorbance was recorded at 560 nm. Various concentrations of hydroxyproline were used as standards, and the results were calculated as μg/mg wet tissue. All morbidities and mortalities throughout the procedures were recorded.

### Statistical analysis

All groups were compared in terms of PtO_2_ levels before, right after, and 30 days after gastrectomy; burst pressure of the gastrectomy line; and hydroxyproline level at the EGJ. Comparisons of PtO_2_ measurements, gastrectomy line burst pressures, EGJ tissue hydroxyproline levels, and change ratios of PtO_2_ levels among the four study groups were analyzed with Kruskal-Wallis test. For two-group comparisons (12-Fr group vs 8-Fr group) of the same variables, Mann-Whitney U test was used to test for statistical significance. The PtO_2_ levels were compared within the same group before, right after, and 30 days after gastrectomy by using Friedman test. When the p value from the Friedman test statistics was statistically significant, multiple comparison test was used to determine which PtO_2_ differs (12). Differences were considered to be statistically significant if p values were <0.05. Statistical Package for Social Sciences 16.0 for Windows (IBM Corporation) was used for statistical evaluation.

## RESULTS

Sleeve gastrectomy was performed on 32 rats, which were divided into four groups. The rats were followed up for 30 days postoperatively. During the study, mortality was detected in two of 32 rats (6.25%). One of the rats belonged to group 2 and died due to gastric leak. No sign of gastric leak was detected at the necropsy of the other rat belonging to group 3. Gastric leak was detected in two of 32 rats (6.25%). One of the rats died on the 3^rd^ day after the surgery (rat in group 2, as mentioned above). Gastric leak in the other rat belonging to group 4 was detected on the 30^th^ day following the laparotomy. This rat was excluded from study. Well-bordered abscesses were determined in the two rats, and fistulas between the stomach and those abscesses were seen during burst pressure measurements.

**Figure 1:**
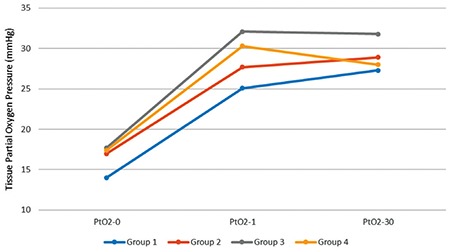
Changes in tissue PtO_2_ levels among the groups. *PtO_2_-0: tissue partial oxygen pressure level before gastrectomy; PtO_2_-1: tissue partial oxygen pressure level right after gastrectomy; PtO_2_-30: tissue partial oxygen pressure level 30 days after gastrectomy*

Tissue partial oxygen pressure at the EGJ was measured before gastrectomy (PtO_2_-0), right after gatrectomy (PtO_2_-1), and on the 30^th^ day following the laparotomy (PtO_2_-30). These values are presented in Table 1, and the corresponding changes are depicted in Figure 1. PtO_2_-0, PtO_2_-1, and PtO_2_-30 values were not significantly different among the four groups. When PtO_2_-0, PtO_2_-1, and PtO_2_-30 values were compared within the same group, statistically significant differences were detected in group 1 (p=0.002), group 3 (p=0.005), and group 4 (p=0.006) but not in group 2 (p=0.05) (Table 1). The levels of tissue partial oxygen pressure right after gastrectomy and 30 days after the gastrectomy significantly increased compared with the level before gastrectomy in groups 1, 3, and 4 (p<0.001 for all comparisons). Moreover, the change ratios in PtO_2_-0 to PtO_2_-1, PtO_2_-0 to PtO_2_-30, and PtO_2_-1 to PtO_2_-30 levels were not significantly different among the four groups (Table 2). We also compared PtO_2_ levels between 12-Fr groups (groups 1 and 3 combined) and 8-Fr groups (groups 2 and 4 combined). The change ratios PtO_2_-0 to PtO_2_-1, PtO_2_-0 to PtO_2_-30, and PtO_2_-1 to PtO_2_-30 levels were not significantly different between the two groups (Table 3).

The burst pressure of the gastrectomy line was similar among the four groups (p=0.48) (Table 4). Although 12-Fr groups showed slightly higher burst pressure levels (262.7±51.9 mmHg) than 8-Fr groups (groups 2 and 4 combined) (245.3±68.2 mmHg), the difference was not statistically significant (p=0.48). No significant differences in tissue hydroxyproline levels at the EGJ were found when the four groups were compared with each other separately (p=0.92) and when they are compared as combined 12-Fr and 8-Fr groups (p=0.59) (Table 4).

## DISCUSSION

Over the past 15 years of sleeve gastrectomy experience, some technical details, such as bougie size, could not be standardized. The lack of this standardization led to reports of highly variable efficacy and complication rates worldwide (7,8,9). For instance, studies assessing the relationship between the use of different bougie sizes and excess weight loss (EWL) in the literature yielded controversial results. Atkins et al. (13) used 40-Fr bougie and obtained better EWL 2 years after the surgery and better resolution of comorbidities compared when 50-Fr was used. Abd Ellatif et al. (14) also reported that better EWL is associated with using bougie with narrow diameter. By contrast, Parikh et al. (15) compared 40-Fr and 50-Fr bougies and reported no differences in weight loss. Cal et al. (16) conducted the only randomized controlled trial that investigated the relationship between weight loss and bougie diameter in 126 patients who underwent LSG; the results showed that the use of different bougie diameters had no effect on EWL after LSG. Despite the contradicting findings in the literature, surgeons have increasingly used narrow bougies to decrease the risk of weight regain. Previous studies also determined whether using large bougie sizes can decrease the leak rate at the gastrectomy line. A large systematic review including 4999 patients demonstrated that large bougie sizes were associated with a significant decrease in leak incidence and lack of changes in weight loss (17). Another meta-analysis including 9991 patients who underwent LSG suggested that utilizing bougie size ≥40-Fr may decrease leak without impacting EWL% (18). The high leak rates observed when using narrow bougie could be attributed to increased intragastric pressure and wall tension and ischemia in the staple line (7,17). However, few studies analyzed how gastric blood flow and tissue perfusion change after sleeve gastrectomy. Gomes et al. (19) evaluated gastric fundus ischemia caused by the sectioning of the short gastric, left gastric, and left gastro-omental arteries by using fluorescein testing and morphometric image analysis in mongrel dogs. This study was designed to investigate the causes of leak after transhiatal subtotal esophagectomy with esophagogastric reconstruction. The results demonstrated significant reduction in blood circulation on the anterior side of the gastric fundus but could not be correlated with those of the present study, where short gastric and left and right gastro-omental arteries are ligated during sleeve gastrectomy and the left gastric artery is preserved. Saber et al. (20) evaluated the gastric wall perfusion of 205 patients in five gastric regions (the angle of his, greater curvature, lesser curvature, incisura angularis, and mid gastric antrum) by using computed tomography scan perfusion index. They found that gastric perfusion at the angle of his was significantly lower than that in the other gastric regions; interestingly, gastric perfusion in all regions was significantly lower in obese patients compared with non-obese patients. Another interesting finding in this study is the presence of better gastric perfusion in hypertensive patients compared with non-hypertensive patients. The authors concluded that gastric leakage in obese patients following sleeve gastrectomy could be attributed to decreased blood supply at the angle of his. However, Natoudi et al. (21) reported contradictory findings. In their study on 12 Landrace swine, they evaluated the effect of ischemia and intraluminal pressure on leak occurrence. They measured lactic acid, glycerol, and pyruvate levels by using microdialysis technique at the EGJ and pylorus before and after the operation to monitor gastric ischemia. They detected increased lactic acid levels and lactate/pyruvate ratio at the EGJ after operation, but no significant difference in these levels was detected between the EGJ and pylorus. They stated no evidence of increased ischemia in association with leakage at the EGJ after sleeve gastrectomy. These results are consistent with the findings of the present study, in which the hypothesis states that the use of narrow bougies is associated with increased risk of leakage due to tissue ischemia and impaired wound healing. We analyzed PtO_2_ and tissue hydroxyproline levels at the EGJ. The PtO_2_ level at the EGJ did not decrease but increased after the surgery in both 8-Fr and 12-Fr groups. Additionally, no significant differences in hydroxyproline levels were detected between 8-Fr and 12-Fr groups. We believe that these results could be due to maintaining the same blood flow to a relatively small residual gastric tissue after the operation, resulting in increased blood supply per unit of tissue. Furthermore, increased PtO_2_-1 levels may be due to the inflammatory response to surgical stress and the release of free oxygen radicals. However, persisting PtO_2_ level on the 30^th^ day supports the theory of increased blood flow per unit of tissue. Thus, the findings of our study are counter evidence to the “impaired blood supply theory” in leak occurrence after sleeve gastrectomy. Given that this work was not designed for investigating the causes of increased tissue oxygenation, further experimental studies must be performed on this particular topic to reveal concrete evidence. Several studies evaluated “whether or not the use of fibrin sealant on gastrectomy line decreases the leak rate.” Some scholars reported the positive effect of fibrin sealant use on leakage prevention (22,23,24). Sapala et al. (22) suggested that fibrin sealant application may also contribute to “leak prophylaxis”. By contrast, a comprehensive meta-analysis demonstrated that several different products, including fibrin sealant, which were used to prevent staple line leakage following sleeve gastrectomy, were helpful for controlling staple line bleeding but not for leakage prevention (18). In the present work, we found no significant effects of fibrin sealant on burst pressure and hydroxyproline levels at the EGJ. We conclude that the use of narrow bougies and fibrin sealant does not affect tissue perfusion and wound healing after sleeve gastrectomy.

## Figures and Tables

**Table 1:**
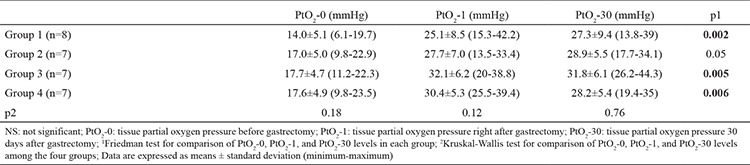
Tissue partial oxygen pressure levels in groups

**Table 2:**
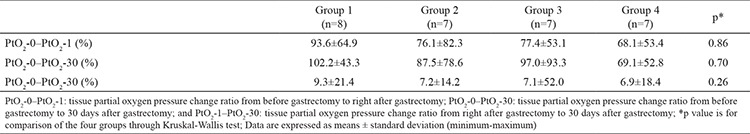
Tissue partial oxygen pressure change ratios in groups

**Table 3:**
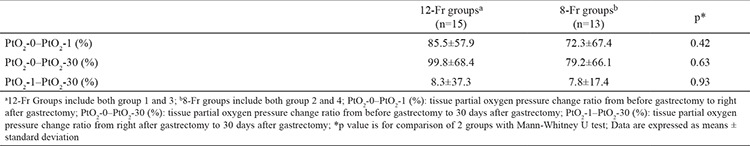
Tissue partial oxygen pressure change ratios by bougie size in groups

**Table 4:**
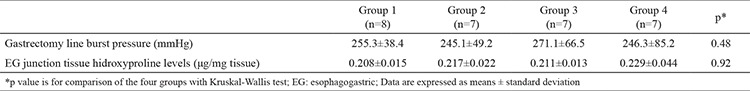
Gastrectomy line burst pressure and esophagogastric junction tissue hydroxyproline levels in groups

## References

[1] ([cited 2016 December 9]). World Health Organization (WHO), "Data and Statistics on Obesity".

[2] Angrisani L, Santonicola A, Iovino P, Formisano G, Buchwald H, Scopinaro N (2015). Bariatric Surgery Worldwide 2013. Obes Surg.

[3] Regan JP, Inabnet WB, Gagner M, Pomp A (2003). Early experience with two-stage laparoscopic Roux-en-Y gastric bypass as an alternative in the super-super obese patient. Obes Surg.

[4] Lim DM, Taller J, Bertucci W, Riffenburgh RH, O'Leary J, Wisbach G (2014). Comparison of laparoscopic sleeve gastrectomy to laparoscopic Roux-en-Y gastric bypass for morbid obesity in a military institution. Surg Obes Relat Dis.

[5] Zhang N, Maffei A, Cerabona T, Pahuja A, Omana J, Kaul A (2013). Reduction in obesity-related comorbidities: is gastric bypass better than sleeve gastrectomy?. Surg Endosc.

[6] Genc V, Sulaimanov M, Kırımker EO, Sevim Y, Ensari C (2015). The use of porcine acellular dermal matrix for management of gastrocutaneous fistula after laparoscopic sleeve gastrectomy. J Laparoendosc Adv Surg Tech A.

[7] Aurora AR, Khaitan L, Saber AA (2012). Sleeve gastrectomy and the risk of leak: a systematic analysis of 4.888 patients.. Surg Endosc.

[8] Tan JT, Kariyawasam S, Wijeratne T, Chandraratna HS (2010). Diagnosis and management of gastric leaks after laparoscopic sleeve gastrectomy for morbid obesity. Obes Surg.

[9] van Rutte PW, Naagen BJ, Spek M, Jakimowicz JJ, Nienhuijs SW (2015). Gastric Wall Thickness in Sleeve Gastrectomy Patients: Thickness Variation of the Gastric Wall. Surg Technol Int.

[10] Kuzu MA, Köksoy C, Kale IT, Tanik A, Terzi C, Elhan AH (1998). Reperfusion injury delays healing of intestinal anastomosis in a rat. Am J Surg.

[11] Jamall IS, Finelli VN, Que Hee SS (1981). A simple method to determine nanogram levels of 4-hydroxyproline in biological tissues. Anal Biochem.

[12] Sidney Siegel NJCJ (1988). The Friedman Two-Way Analysis of Variance by Ranks. in Nonparametric Statistics for the Behavioral Sciences.

[13] Atkins ER, Preen DB, Jarman C, Cohen LD (2012). Improved obesity reduction and co-morbidity resolution in patients treated with 40-French bougie versus 50-French bougie four years after laparoscopic sleeve gastrectomy. Analysis of 294 patients. Obes Surg.

[14] Abd Ellatif ME, Abdallah E, Askar W, Thabet W, Aboushady M, Abbas AE, et al (2014). Long term predictors of success after laparoscopic sleeve gastrectomy. Int J Surg.

[15] Parikh M, Gagner M, Heacock L, Strain G, Dakin G, Pomp A (2008). Laparoscopic sleeve gastrectomy: does bougie size affect mean %EWL? Short-term outcomes. Surg Obes Relat Dis.

[16] Cal P, Deluca L, Jakob T, Fernández E (2016). Laparoscopic sleeve gastrectomy with 27 versus 39 Fr bougie calibration: a randomized controlled trial. Surg Endosc.

[17] Yuval JB, Mintz Y, Cohen MJ, Rivkind AI, Elazary R (2013). The effects of bougie caliber on leaks and excess weight loss following laparoscopic sleeve gastrectomy. Is there an ideal bougie size?. Obes Surg.

[18] Parikh M, Issa R, McCrillis A, Saunders JK, Ude-Welcome A, Gagner M (2013). Surgical strategies that may decrease leak after laparoscopic sleeve gastrectomy: a systematic review and meta-analysis of 9991 cases. Ann Surg.

[19] Gomes M, Ramacciotti E, Miranda F Jr, Henriques AC, Fagundes DJ (2009). Vascular flow of the gastric fundus after arterial devascularization: an experimental study. J Surg Res.

[20] Saber AA, Azar N, Dekal M, Abdelbaki TN (2015). Computed tomographic scan mapping of gastric wall perfusion and clinical implications. Am J Surg.

[21] Natoudi M, Theodorou D, Papalois A, Drymousis P, Alevizos L, Katsaragakis S, et al (2014). Does tissue ischemia actually contribute to leak after sleeve gastrectomy? An experimental study. Obes Surg.

[22] Sapala JA, Wood MH, Schuhknecht MP (2004). Anastomotic leak prophylaxis using a vapor-heated fibrin sealant: report on 738 gastric bypass patients. Obes Surg.

[23] Liu CD, Glantz GJ, Livingston EH (2003). Fibrin glue as a sealant for high-risk anastomosis in surgery for morbid obesity. Obes Surg.

[24] Nguyen NT, Nguyen CT, Stevens CM, Steward E, Paya M (2004). The efficacy of fibrin sealant in prevention of anastomotic leak after laparoscopic gastric bypass. J Surg Res.

